# Crystal structure and spectroscopic properties of aqua­dichlorido­{1,1′-[(pyridine-2,6-diyl-κ*N*)bis(methyl­ene)]bis­(4-butyl-4,5-di­hydro-1*H*-1,2,4-triazole-5-thione-κ*N* ^2^)}cobalt(II)

**DOI:** 10.1107/S2056989020013547

**Published:** 2020-10-16

**Authors:** John R. Miecznikowski, Jerry P. Jasinski, Tyler J. Ostrowski, Kendra R. Landy, Sheila C. Bonitatibus, Allison N. Smolinsky, Natalia R. Bertolotti

**Affiliations:** a Department of Chemistry and Biochemistry, Fairfield University, 1073 North Benson Road, Fairfield, CT 06824, USA; b Department of Chemistry, Keene State College, 229 Main Street, Keene, NH 03435, USA

**Keywords:** crystal structure, electrospray mass spectrum, NMR, cobalt(II), pincer complex

## Abstract

The structure of the title compound, [CoCl_2_(C_19_H_27_N_7_S_2_)(H_2_O)], at 173 K has monoclinic (*C*2/*c*) symmetry. We report here the synthesis, single-crystal structure, electrospray mass spectrum and NMR spectroscopy of a new six-coordinate cobalt(II) pincer complex. The pincer ligand, in this complex, which is novel, coordinates *via* three nitro­gen atoms (two triazole and one pyridine). The ligand is ambidentate and can coordinate *via* three nitro­gen atoms or two sulfur and one nitro­gen atoms. The cobalt(II) metal center has pseudo-octa­hedral geometry and based on the single-crystal structure, the pincer ligand coordinates in a meridional fashion with the metal and adjacent six-membered ring ligands all in a similar plane and forming two slightly distorted boat configurations. The other two coordinated monodentate ligands are one water mol­ecule and two chloride ions with four cobalt(II) complexes in the unit cell. The asymmetric unit of the complex is comprised of half the pyridine ring and water mol­ecule with the Co^II^ atom at the center of the pincer situated about a twofold axis. The Co—N, Co—O, and Co—Cl bond lengths are consistent with single bonds. In the crystal, the complex forms a three-centre bifurcated weak hydrogen-bonding inter­action with a chlorine ion, forming one inter­molecular inter­action with the pincer group and a water mol­ecule and a second intra­molecular inter­action with a C—H group within the pincer group. Crystal packing is also highlighted with *C*_2_^2^(6)>*a*<*a* infinite chains forming along [001] supported by *R*_2_^2^(8)>*a*>*a* ring motifs, forming a three-dimensional supra­molecular network structure. While some stacking of the pyridine rings in the unit cell is observed, there are no relevant π–π inter­actions in the crystal packing. The ^1^H and ^13^C{^1^H} NMR spectra of the complex are consistent with a plane of symmetry being present. The electrospray mass spectrum, which was collected in positive ion mode, showed the loss of one water mol­ecule and one chloride ligand from the complex. In the future, we plan to screen this cobalt(II) complex for electrocatalysis reactivity.

## Abstract 2

We report here the synthesis, single-crystal structure, electrospray mass spectrum and NMR spectroscopy of a new six-coordinate cobalt(II) pincer complex. The pincer ligand, in this complex, which is novel, coordinates *via* three nitro­gen atoms (two triazole and one pyridine). The ligand is ambidentate and can coordinate *via* three nitro­gen atoms or two sulfur and one nitro­gen atoms. The cobalt(II) metal center has pseudo-octa­hedral geometry and based on the single-crystal structure, the pincer ligand coordinates in a meridional fashion with the metal and adjacent six-membered ring ligands all in a similar plane and forming two slightly distorted boat configurations

## Chemical context   

**Figure 1:**
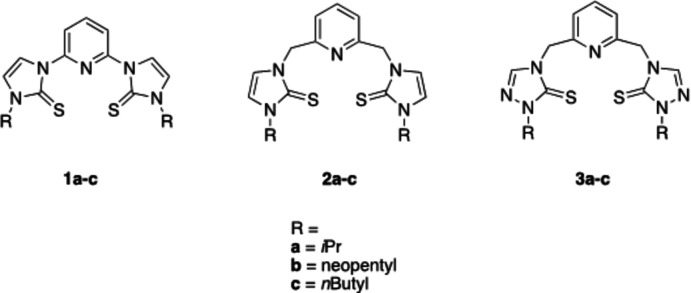
SNS ligand precursors prepared by Miecznikowski *et al.* (2011 ▸, 2012 ▸).

Pincer ligands are tridentate ligands that coordinate to metal centers either in a meridional or facial manner (Peris & Crabtree, 2018 ▸; Gunanathan & Milstein, 2014 ▸). The resulting pincer complexes are robust and have been utilized as catalysts in a variety of reactions (Szabó & Wendt, 2014 ▸). Pincer complexes can be prepared using a wide range of metal centers. The donor atoms of the pincer ligand to the metal can be carbon, oxygen, nitro­gen, phospho­rous, or sulfur (Peris & Crabtree, 2018 ▸). Pincer ligand precursors can be tuned electronically by including electron-withdrawing or electron-donating groups, and sterically by including bulky substituents (van Koten & Milstein, 2013 ▸; van Koten & Gossage, 2015 ▸). Previously, Miecznikowski and co-workers prepared tridentate pincer ligand precursors with sulfur, nitro­gen and sulfur donor atoms (Miecznikowski *et al.*, 2011 ▸, 2012 ▸) (Fig. 1 ▸). The pincer ligand precursors were metallated with zinc(II)chloride in order to prepare zinc(II) model complexes of liver alcohol de­hydrogenase (Miecznikowski *et al.*, 2011 ▸, 2012 ▸) (see reaction scheme below). In 2012, Miecznikowski and co-workers reported the preparation of a pincer ligand precursor based on a bis-triazole starting material that could coordinate *via* sulfur, nitro­gen, and sulfur donor atoms or *via* three nitro­gen donor atoms (Miecznikowski *et al.*, 2012 ▸). It was reported that the novel ambidentate tridentate pincer ligand precursor was metallated with ZnCl_2_ to give a new tridentate NNN-bound pincer zinc(II) pincer complex: di­chloro­(η3-*N*,*N*,*N*)-[2,6-bis­(3-[*N*-but­yl]triazol-5-thione-1-yl)]pyridine­zinc(II), [(NNN)ZnCl_2_] (Fig. 1 ▸).
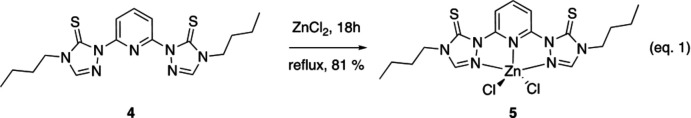


**Figure 2:**
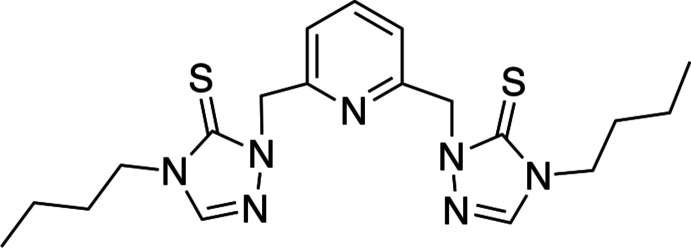
Structure of the ambidentate pincer ligand precursor.

In this study, our aim was to prepare a cobalt(II) pincer complex that contained a pincer ligand precursor with methyl­ene moieties connecting each triazole substituent to the pyridine in the pincer ligand precursor (Fig. 2 ▸). We wondered if the cobalt(II) metal center would coordinate to the pincer ligand *via* three nitro­gen atoms as observed for the zinc(II) complex or *via* sulfur, nitro­gen, and sulfur donor atoms. In this communication, we report the preparation, spectroscopic characterization, electrospray mass spectrometry, and single crystal structure of a cobalt(II) pincer complex that contains an ambidentate ligand (Fig. 2 ▸).

## Structural commentary   

**Figure 3:**
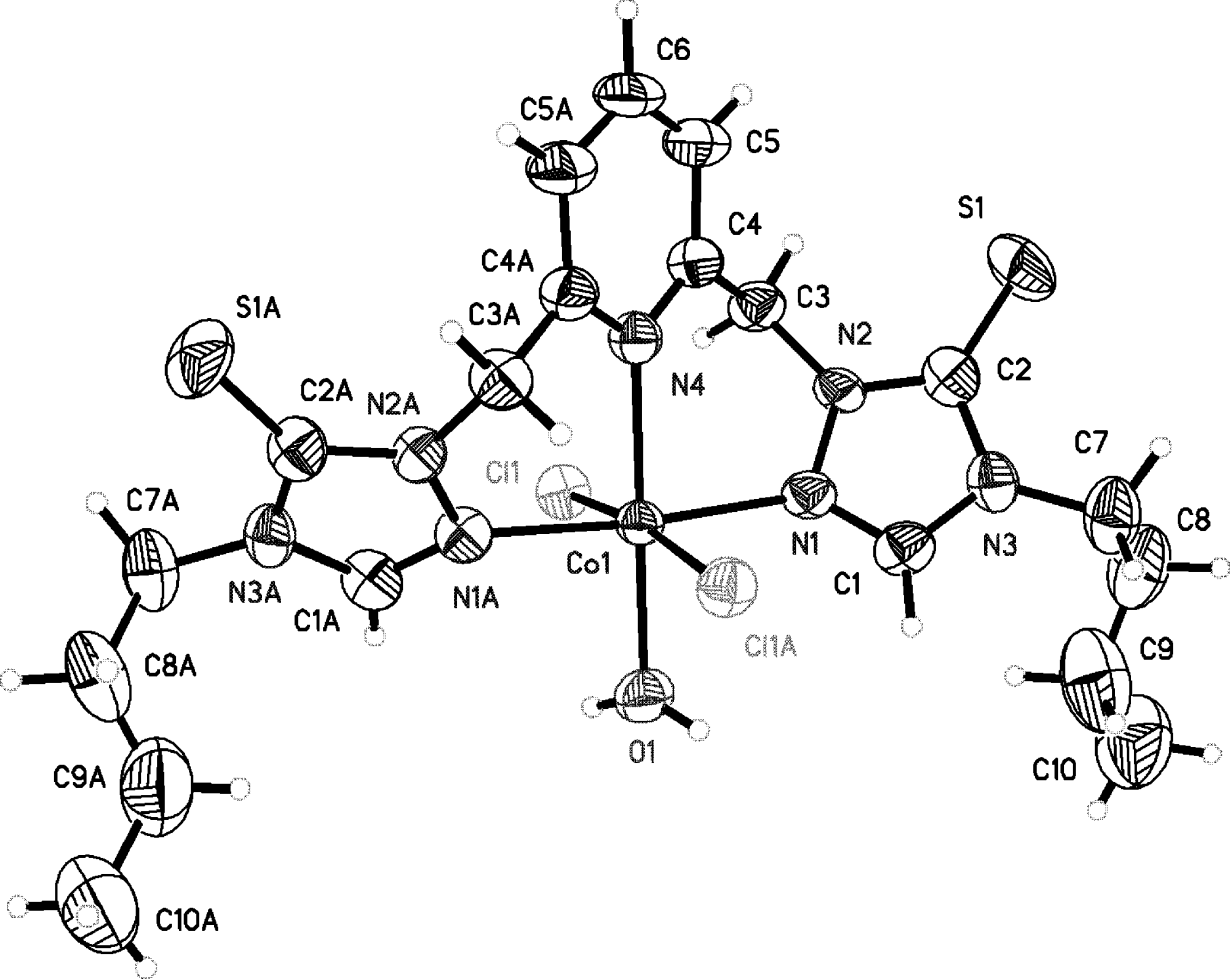
A view of the mol­ecular structure of C_19_H_29_Cl_2_CoN_7_OS_2_, **8**, showing the atom-labeling scheme and displacement ellipsoids drawn at the 30% probability level. The mol­ecule crystallizes in the *C*2/*c* space group with a twofold rotation axis perpendicular to a *c*-glide plane along the center of the pincer ligand through to the metal ion transforming the two asymmetric units into the complete complex and containing four cobalt(II) complexes per unit cell. Atoms with the suffix A are generated by the symmetry operation 1 − *x*, *y*, −*z* + 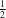.

We report here the synthesis, single crystal structure, electrospray mass spectrum and NMR spectroscopy of a new six-coordinate cobalt(II) pincer complex, C_19_H_29_Cl_2_CoN_7_OS_2_, at 173 K whose structure has monoclinic (*C*2/*c*) symmetry (Fig. 3 ▸). The pincer ligand, in this complex, which is novel, coordinates *via* three nitro­gen atoms (two triazole and one pyridine). The ligand is ambidentate and can coordinate *via* three nitro­gen atoms or two sulfur and one nitro­gen atom. The cobalt(II) metal center has a pseudo-octa­hedral geometry and based on the single crystal structure, the pincer ligand coordinates in a meridional fashion with the metal and adjacent six-membered ring ligands all in a similar plane and forming two slightly distorted boat configurations [Co1/N2/N2/C3/C4/N4: *Q*1 = 0.743 (6) Å, θ = 89.9°, φ = 345.8 (5)°; Co1/N4/C4A/C3A/N2A/N1A (atoms with the suffix A are generated by the symmetry operation 1 − *x*, *y*, −*z* + 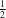): *Q*2 = 0.743 (6) Å, θ = 90.1(5°, φ = 185.3 (5)°} (Cremer & Pople, 1975 ▸). The other two coordinated monodentate ligands are one water mol­ecule and two chloride ions with four cobalt(II) complexes in the unit cell. The asymmetric unit of the complex is comprised of half the pyridine ring and water mol­ecule with the Co^II^ atom at the center of the pincer situated about a twofold axis. The Co—N, Co—O, and Co—Cl bond lengths are consistent with single bonds. The cobalt–nitro­gen (triazole) and cobalt–nitro­gen (pyridine) bond lengths are comparable to those previously reported [triazole Co—N = 2.127 (2) and 2.093 (2) Å; pyridine Co—N= 2.187 (3) Å (Fang *et al.*, 2019 ▸)]. The Co—O(water) bond length is comparable to previously reported values [2.09 (3) Å; See *et al.*, 1998 ▸]. The cobalt–chloride bond lengths are longer than previously reported (2.31 Å; Di Vaira & Orioli, 1965 ▸). The C=S bond length of 1.655 (7) Å is more consistent with a carbon–sulfur double bond (1.61 Å; Trzhtsinskaya & Abramova, 1991 ▸).

## Supra­molecular features   

**Figure 4:**
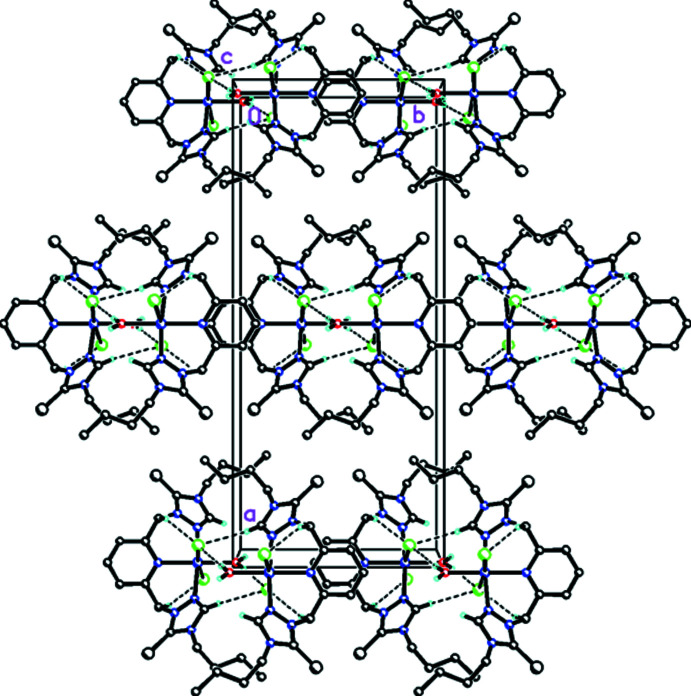
A view of the packing and the unit cell along the *c* axis for the title complex, **8**. Dashed lines indicate 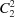
(6)>*a*<*a* infinite chains forming along (001) and 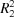
(8)>*a*>*a* ring motifs forming a three-dimensional supra­molecular network structure. Stacking of the pyridyl rings face-to-face along the *c* axis is observed.

In the crystal, the complex forms a three-centre bifurcated weak hydrogen-bonding inter­action (Table 1 ▸) with a chlorine ion, forming one inter­molecular inter­action with the pincer group and a water mol­ecule and a second intra­molecular inter­action with a C—H group within the pincer group. The crystal packing is also highly supported by 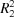
(8)>*a*>*a* ring motifs, forming a three-dimensional supra­molecular network structure (Fig. 4 ▸). While some stacking of the pyridine rings in the unit cell is observed, there are no relevant π–π inter­actions or classical hydrogen bonds in the crystal packing. The ^1^H and ^13^C{^1^H} NMR spectra of the complex are consistent with a plane of symmetry being present. The electrospray mass spectrum, which was collected in positive ion mode, showed the loss of one water mol­ecule and one chloride ligand from the complex (see Fig. S1 in the supporting information). In the future, we plan to screen this cobalt(II) complex for electrocatalysis reactivity.

## Database survey   

The NNN pincer ligand precursor used in this study is novel. A related NNN pincer ligand, **4**, has only been metallated with ZnCl_2_ to afford a five-coordinate zinc(II) complex (Miecznikowski *et al.*, 2013 ▸). To the best of our knowledge (following a search using Sci-Finder Scholar) no other metal complexes that contain this ligand precursor have been reported in the literature.

## Synthesis and crystallization   

The preparation of the title complex, **8**, and the corres­ponding ligand precursors **6** and **7** were accomplished according to the scheme below. The precursor for complex **6** has been reported previously (Guino-o *et al.*, 2015 ▸).
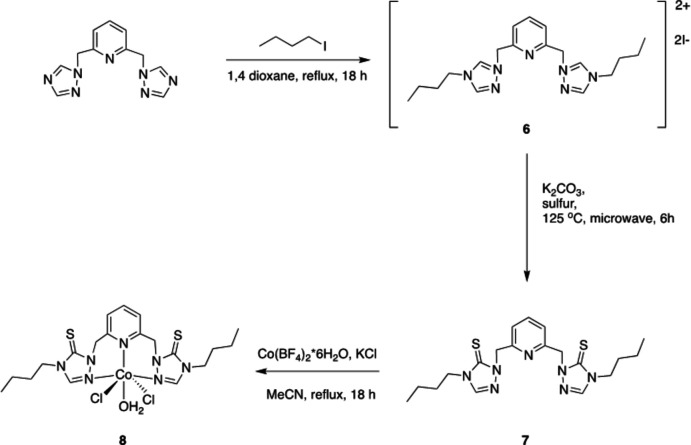


**2,6-Bis[(4-butyl-4** ***H*** **-1,2,4-triazol-1-yl)meth­yl]pyridine diiodide** (Miecznikowski *et al.*, 2011 ▸): In a 100 ml round-bottom flask, 3.0501 g (0.0126 mol) of 2,6-bis­[(1*H*-1,2,4-triazol-1-yl)meth­yl]pyridine was dissolved in 25 ml of 1,4 dioxane. To this solution 13.958 g (0.0759 mol) of iodo­butane were added. This solution was heated at reflux for 18 h. After allowing the solution to cool, the mother liquor was deca­nted off. The precipitate was dissolved in minimal methanol and transferred to a round-bottom flask. The solvent was then removed under reduced pressure. Yield: 3.85 g (0.00748 mol) (59.4% yield).

The product was characterized using ^1^H and ^13^C{^1^H} NMR spectroscopy.

**^1^H NMR (DMSO-** ***d*** **_6_, 400 Mhz)**: δ 10.32 (*s*, 2H, triazole, CH), 9.30 (*s*, 2H, triazole, CH), 7.97 (*m*, 1H, pyridine CH), 7.52 (*m*, (2H, pyridine CH), 5.75 (*s*, 4H, CH_2_ linker), 4.32 (*m*, 4H, *n*-butyl CH_2_), 1.85 (*m*, 4H, *n*-butyl CH_2_), 1.32 (*m*, 4H, *n*-butyl CH_2_), 0.92 (*m*, 6H, *n*-butyl CH_3_). ^13^C {^1^H} NMR (DMSO-*d*_6_, 100 Mhz), δ 152.59 (pyridine C), 144.91 (triazole CH), 143.51 (triazole CH), 138.95 (pyridine CH), 122.85 (pyridine CH), 55.74 (CH_2_ linker), 47.42 (*n*-butyl CH_2_), 30.82 (*n*-butyl CH_2_), 18.79 (*n*-butyl CH_2_), 13.34 (*n*-butyl CH_3_).

**2,2′-[Pyridine-2,6-diylbis(methyl­ene)]bis­(4-butyl-1,2,4-triazole-3-thione)** (Miecznikowski, 2012 ▸): In a 35 mL microwave reactor vessel, 0.2694 g (5.228 × 10 ^−4^ mol) of **6** were dissolved in 15 mL of MeCN. To this solution, 0.800 g (0.0249 mol) of sulfur and 0.2162 g (0.001564 mol) of potassium carbonate were added. This mixture was heated at 398 K for 6 h in the microwave reactor. After the reaction was complete, the undissolved solids were removed by vacuum filtration and the remaining solvent was removed under reduced pressure. The product was purified by dissolving the product in CH_2_Cl_2_ and filtering it through an alumina column to removed undissolved sulfur. Mass product = 0.0934 g (2.24 x 10 ^−4^ mol) (42.8% yield).

The product was characterized using ^1^H and ^13^C{^1^H} NMR spectroscopy. The key feature of the NMR is that one of the acidic protons of the triazole, δ 10.32 in **6** was absent in the product and was presumably replaced with the thione moiety. There was also considerable shifting, to lower ppm values, of the aromatic, methyl­ene and *n*-butyl CH_2_ proton resonances in **7** compared to the starting material **6**.

**^1^H NMR (DMSO-** ***d*** **_6_, 400 MHz)**: δ 8.65 (*s*, 2H, triazole, CH), 7.75 (*m*, 1H, pyridine CH), 7.02 (*s*, 2H, pyridine CH), 5.41 (*s*, 4H, CH_2_ linker), 3.98 (*m*, 4H, *n*-butyl CH_2_), 1.72 (*m*, 4H, *n*-butyl CH_2_), 1.29 (*m*, 4H, *n*-butyl CH_2_), 0.92 (*m*, 6H, *n*-butyl CH_3_). ^13^C {1H} NMR (DMSO-*d*_6_, 100 MHz), δ 166.03 (C=S), 155.02 (pyridine C), 141.41 (triazole CH), 137.92 (pyridine CH), 120.23 (pyridine CH), 53.03 (CH_2_ linker), 45.14 (*n*-butyl CH_2_), 29.91 (*n*-butyl CH_2_), 19.04 (*n*-butyl CH_2_), 13.47 (*n*-butyl CH_3_).

**Aqua­dichloro-(n3-** ***N***,***N***,***N*** **)-[2,6-diylbis(methyl­ene)bis­(4-[** ***N*** **-but­yl]triazol-5-thione-1-yl)]pyridine­cobalt(II) [C_19_H_29_Cl_2_N_7_OS_2_Co]**: In a 100mL round-bottom flask, 0.0934 g (2.24 × 10^−4^ mol) of (C_19_H_27_N_7_S_2_) were combined with 0.076 g (2.2 × 10^−4^ mol) of cobalt(II)tetra­fluoro­borate [Co(BF_4_)_2_·6H_2_O] and combined with 0.0333 g (4.43 × 10^−4^ moles) of potassium chloride (KCl) and dissolved in 10 mL of aceto­nitrile. The solution was refluxed for 20 h. The following day, the solution was filtered to remove undissolved material and the solvent was removed under reduced pressure. Yield: 0.162 grams (qu­anti­tative). Purple needle-shaped crystals suitable for X-ray diffraction were grown by a slow vapor diffusion of diethyl ether in to an aceto­nitrile solution containing the cobalt complex.

**Analysis calculated for [C_19_H_29_Cl_2_CoN_7_S_2_]·H_2_O:** (583.46): C, 39.11; H, 5.36; N, 16.80. Found: C, 39.33; H, 5.07; N, 17.11.

**^1^H NMR (DMSO-** ***d*** **_6_, 400 MHz)** δ 8.61 (*s*, 2H, triazole, CH), 7.71 (*t*, 1H, pyridine CH), 6.91 (*d*, 2H pyridine CH), 5.37 (*s*, 4H, CH_2_ linker), 3.95 (*m*, 4H, *n*-butyl CH_2_), 1.70 (*m*, 4H, *n*-butyl CH_2_), 1.26 (*m*, 4H, *n*-butyl CH_2_), 0.88 (*t*, 6H, *n*-butyl CH_3_). ^13^C {^1^H} NMR (DMSO-*d*_6_, 100 MHz), δ 164.95 (C=S), 153.93 (pyridine C), 140.47 (triazole CH), 136.91 (pyridine CH), 119.19 (pyridine CH), 51.98 (CH_2_ linker), 44.13 (*n*-butyl CH_2_), 28.87 (*n*-butyl CH_2_), 18.01 (*n*-butyl CH_2_), 12.47 (*n*-butyl CH_3_).

The ^1^H NMR and ^13^C{^1^H} spectrum of the complex was acquired in DMSO-*d*_6_. The NMR spectrum was consistent with the title complex. There was not considerable shifting of the proton resonances compared to the starting bis-thione precursor. In the ^13^C{1H} NMR spectrum, the C=S resonance shifted about δ 1 ppm to a lower chemical shift value.

The cyclic voltammograms of **7** and **8** are given in Figs. S1 and S2, respectively, in the supporting information. In both **7** and **8** the supporting electrolyte is 0.2 *M* tetrabutylammonium tetrafluoroborate. The reference electrode is Ag wire, the working electrode is glassy carbon and the counter electrode is a platinum wire. The scan rate is 100 mV s-1.

**Electronic absorption spectrum of 8 in aceto­nitrile (1.89 m** ***M*** **)** (see Fig. S4 in the supporting information): UV–Visible data: λ (nm), (∊ (M^−1^cm^−1^) (1.89 m*M* in MeCN) 234.00 (2480); 244.00 (2480); 368.00 (158); 574.00 nm (sh) (386); 589.00 (428); 682.00 (648).

## Refinement   

Crystal data, data collection and structure refinement details are summarized in Table 2 ▸. H atoms were positioned geometrically (O—H = 0.84, C—H = 0.95–0.99 Å) and refined as riding with *U*_iso_(H) = 1.2*U*_eq_(C) or 1.5*U*_eq_(O, C-meth­yl).

## Supplementary Material

Crystal structure: contains datablock(s) I. DOI: 10.1107/S2056989020013547/jy2002sup1.cif

Structure factors: contains datablock(s) I. DOI: 10.1107/S2056989020013547/jy2002Isup2.hkl

Supplementary figures. DOI: 10.1107/S2056989020013547/jy2002sup2.pdf

CCDC reference: 2036378

Additional supporting information:  crystallographic information; 3D view; checkCIF report

## Figures and Tables

**Table 1:** Hydrogen-bond geometry (Å, °)

*D*—H⋯*A*	*D*—H	H⋯*A*	*D*⋯*A*	*D*—H⋯*A*
O1—H1⋯Cl1^i^	0.84	2.24	3.075 (4)	171
C1—H1*A*⋯O1^ii^	0.95	2.69	3.429 (7)	135
C3—H3*A*⋯Cl1	0.99	2.55	3.360 (8)	138

Symmetry codes: (i) 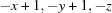; (ii) 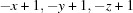.

**Table 2:** Experimental details

Crystal data	
Chemical formula	[CoCl_2_(C_19_H_27_N_7_S_2_)(H_2_O)]
*M*_r_	565.44
Crystal system, space group	Monoclinic, *C*2/*c*
Temperature (K)	173
*a*, *b*, *c* (Å)	26.3412 (8), 11.4270 (3), 8.6226 (3)
β (°)	90.465 (3)
*V* (Å^3^)	2595.31 (13)
*Z*	4
Radiation type	Cu *K*α
μ (mm^−1^)	8.80
Crystal size (mm)	0.32 × 0.24 × 0.1

Data collection	
Diffractometer	Rigaku Oxford Diffraction Gemini Eos
Absorption correction	Multi-scan (*CrysAlis PRO*; Rigaku OD, 2015 ▸)
*T*_min_, *T*_max_	0.050, 1.000
No. of measured, independent and observed [*I* > 2σ(*I*)] reflections	7187, 2483, 1935
*R*_int_	0.077
(sin θ/λ)_max_ (Å^−1^)	0.614

Refinement	
*R*[*F* ^2^ > 2σ(*F* ^2^)], *wR*(*F* ^2^), *S*	0.082, 0.235, 1.07
No. of reflections	2483
No. of parameters	149
H-atom treatment	H-atom parameters constrained
Δρ_max_, Δρ_min_ (e Å^−3^)	1.40, −0.65

Computer programs: *CrysAlis PRO* (Rigaku OD, 2015 ▸), *SHELXT* (Sheldrick, 2015*b* ▸), *SHELXL* (Sheldrick, 2015*a* ▸) and *OLEX2* (Dolomanov *et al.*, 2009 ▸ ▸).

## supplementary crystallographic information

### Crystal data

**Table:**

[CoCl_2_(C_19_H_27_N_7_S_2_)(H_2_O)]	*F*(000) = 1172
*M**_r_* = 565.44	*D*_x_ = 1.447 Mg m^−^^3^
Monoclinic, *C*2/*c*	Cu *K*α radiation, λ = 1.54184 Å
*a* = 26.3412 (8) Å	Cell parameters from 3680 reflections
*b* = 11.4270 (3) Å	θ = 5.1–71.4°
*c* = 8.6226 (3) Å	µ = 8.80 mm^−^^1^
β = 90.465 (3)°	*T* = 173 K
*V* = 2595.31 (13) Å^3^	Plate, violet
*Z* = 4	0.32 × 0.24 × 0.1 mm

### Data collection

**Table:**

Rigaku Oxford Diffraction Gemini Eos diffractometer	1935 reflections with *I* > 2σ(*I*)
Detector resolution: 16.0416 pixels mm^-1^	*R*_int_ = 0.077
ω scans	θ_max_ = 71.3°, θ_min_ = 3.4°
Absorption correction: multi-scan (CrysAlisPro; Rigaku OD, 2015)	*h* = −31→32
*T*_min_ = 0.050, *T*_max_ = 1.000	*k* = −14→13
7187 measured reflections	*l* = −10→5
2483 independent reflections	

### Refinement

**Table:**

Refinement on *F*^2^	Hydrogen site location: mixed
Least-squares matrix: full	H-atom parameters constrained
*R*[*F*^2^ > 2σ(*F*^2^)] = 0.082	*w* = 1/[σ^2^(*F*_o_^2^) + (0.0964*P*)^2^ + 26.3272*P*] where *P* = (*F*_o_^2^ + 2*F*_c_^2^)/3
*wR*(*F*^2^) = 0.235	(Δ/σ)_max_ < 0.001
*S* = 1.07	Δρ_max_ = 1.40 e Å^−^^3^
2483 reflections	Δρ_min_ = −0.65 e Å^−^^3^
149 parameters	Extinction correction: SHELXL2018/1 (Sheldrick 2015b), Fc^*^=kFc[1+0.001xFc^2^λ^3^/sin(2θ)]^-1/4^
0 restraints	Extinction coefficient: 0.0012 (2)
Primary atom site location: dual	

### Special details

**Table:**

Geometry. All esds (except the esd in the dihedral angle between two l.s. planes) are estimated using the full covariance matrix. The cell esds are taken into account individually in the estimation of esds in distances, angles and torsion angles; correlations between esds in cell parameters are only used when they are defined by crystal symmetry. An approximate (isotropic) treatment of cell esds is used for estimating esds involving l.s. planes.

### Fractional atomic coordinates and isotropic or equivalent isotropic displacement parameters (Å^2^)

**Table:**

	*x*	*y*	*z*	*U*_iso_*/*U*_eq_	
Co1	0.500000	0.67881 (12)	0.250000	0.0337 (5)	
Cl1	0.54526 (6)	0.66425 (15)	0.00348 (19)	0.0453 (5)	
S1	0.67541 (8)	0.8989 (2)	0.5267 (3)	0.0709 (7)	
O1	0.500000	0.4946 (6)	0.250000	0.0528 (18)	
H1	0.490570	0.453156	0.174640	0.079*	
N1	0.5694 (2)	0.6878 (5)	0.3822 (6)	0.0404 (13)	
N2	0.5993 (2)	0.7856 (5)	0.3730 (7)	0.0400 (13)	
N3	0.6244 (2)	0.6944 (6)	0.5757 (7)	0.0439 (14)	
N4	0.500000	0.8712 (7)	0.250000	0.0396 (17)	
C1	0.5856 (3)	0.6350 (7)	0.5066 (8)	0.0451 (16)	
H1A	0.571981	0.563686	0.544692	0.054*	
C2	0.6333 (3)	0.7948 (7)	0.4898 (9)	0.0472 (17)	
C3	0.5931 (3)	0.8675 (6)	0.2452 (9)	0.0454 (16)	
H3A	0.594825	0.824119	0.145975	0.054*	
H3B	0.621543	0.924266	0.247439	0.054*	
C4	0.5441 (3)	0.9327 (6)	0.2516 (8)	0.0456 (16)	
C5	0.5448 (4)	1.0553 (7)	0.2553 (11)	0.065 (2)	
H5	0.576185	1.096191	0.261514	0.078*	
C6	0.500000	1.1154 (10)	0.250000	0.076 (4)	
H6	0.500000	1.198536	0.250002	0.092*	
C7	0.6532 (3)	0.6589 (9)	0.7150 (10)	0.065 (2)	
H7A	0.629478	0.621798	0.788644	0.078*	
H7B	0.666974	0.730085	0.765450	0.078*	
C8	0.6957 (4)	0.5769 (11)	0.6865 (13)	0.083 (3)	
H8A	0.717687	0.573444	0.780111	0.100*	
H8B	0.716444	0.606952	0.599897	0.100*	
C9	0.6777 (5)	0.4566 (15)	0.6479 (17)	0.116 (5)	
H9A	0.661282	0.459438	0.544311	0.139*	
H9B	0.651149	0.435140	0.723408	0.139*	
C10	0.7162 (6)	0.3608 (15)	0.6464 (19)	0.125 (5)	
H10A	0.743434	0.381083	0.574431	0.188*	
H10B	0.700015	0.287769	0.612867	0.188*	
H10C	0.730446	0.350724	0.750896	0.188*	

### Atomic displacement parameters (Å^2^)

**Table:**

	*U*^11^	*U*^22^	*U*^33^	*U*^12^	*U*^13^	*U*^23^
Co1	0.0320 (7)	0.0305 (8)	0.0387 (8)	0.000	−0.0011 (6)	0.000
Cl1	0.0447 (9)	0.0495 (10)	0.0417 (9)	0.0014 (7)	0.0047 (7)	−0.0035 (7)
S1	0.0442 (10)	0.0749 (15)	0.0934 (17)	−0.0210 (10)	−0.0088 (10)	−0.0141 (12)
O1	0.079 (5)	0.038 (4)	0.041 (4)	0.000	−0.007 (3)	0.000
N1	0.035 (3)	0.041 (3)	0.045 (3)	−0.008 (2)	0.003 (2)	0.005 (2)
N2	0.032 (3)	0.037 (3)	0.052 (3)	−0.013 (2)	−0.002 (2)	0.004 (2)
N3	0.028 (3)	0.058 (4)	0.046 (3)	−0.002 (2)	−0.001 (2)	−0.001 (3)
N4	0.041 (4)	0.037 (4)	0.041 (4)	0.000	−0.001 (3)	0.000
C1	0.037 (3)	0.047 (4)	0.052 (4)	0.000 (3)	0.009 (3)	0.010 (3)
C2	0.031 (3)	0.053 (4)	0.058 (4)	−0.002 (3)	0.003 (3)	−0.002 (3)
C3	0.041 (4)	0.043 (4)	0.052 (4)	−0.010 (3)	0.005 (3)	0.008 (3)
C4	0.052 (4)	0.040 (4)	0.045 (4)	−0.003 (3)	−0.002 (3)	0.001 (3)
C5	0.072 (5)	0.038 (4)	0.086 (6)	−0.008 (4)	−0.014 (5)	0.006 (4)
C6	0.080 (9)	0.030 (6)	0.119 (12)	0.000	−0.035 (8)	0.000
C7	0.043 (4)	0.096 (7)	0.055 (5)	−0.001 (4)	−0.004 (4)	0.006 (5)
C8	0.057 (5)	0.117 (10)	0.075 (6)	0.012 (6)	−0.011 (5)	0.012 (6)
C9	0.089 (8)	0.154 (14)	0.104 (10)	0.022 (9)	−0.028 (7)	−0.035 (9)
C10	0.106 (10)	0.141 (13)	0.129 (12)	0.026 (10)	−0.028 (9)	0.008 (10)

### Geometric parameters (Å, º)

**Table:**

Co1—Cl1	2.4512 (16)	C3—H3A	0.9900
Co1—Cl1^i^	2.4512 (16)	C3—H3B	0.9900
Co1—O1	2.104 (7)	C3—C4	1.493 (10)
Co1—N1	2.149 (6)	C4—C5	1.401 (11)
Co1—N1^i^	2.148 (6)	C5—H5	0.9500
Co1—N4	2.198 (8)	C5—C6	1.367 (11)
S1—C2	1.655 (7)	C6—H6	0.9500
O1—H1	0.8403	C7—H7A	0.9900
O1—H1^i^	0.8403	C7—H7B	0.9900
N1—N2	1.370 (7)	C7—C8	1.482 (14)
N1—C1	1.300 (9)	C8—H8A	0.9900
N2—C2	1.346 (9)	C8—H8B	0.9900
N2—C3	1.454 (9)	C8—C9	1.491 (18)
N3—C1	1.360 (9)	C9—H9A	0.9900
N3—C2	1.387 (10)	C9—H9B	0.9900
N3—C7	1.472 (10)	C9—C10	1.493 (19)
N4—C4^i^	1.357 (8)	C10—H10A	0.9800
N4—C4	1.357 (8)	C10—H10B	0.9800
C1—H1A	0.9500	C10—H10C	0.9800
			
Cl1^i^—Co1—Cl1	172.22 (10)	N2—C3—C4	112.6 (6)
O1—Co1—Cl1^i^	86.11 (5)	H3A—C3—H3B	107.8
O1—Co1—Cl1	86.11 (5)	C4—C3—H3A	109.1
O1—Co1—N1^i^	92.74 (16)	C4—C3—H3B	109.1
O1—Co1—N1	92.74 (16)	N4—C4—C3	118.8 (6)
O1—Co1—N4	180.0	N4—C4—C5	122.0 (8)
N1^i^—Co1—Cl1^i^	92.61 (15)	C5—C4—C3	119.2 (7)
N1—Co1—Cl1	92.61 (15)	C4—C5—H5	120.3
N1—Co1—Cl1^i^	87.76 (15)	C6—C5—C4	119.3 (9)
N1^i^—Co1—Cl1	87.76 (15)	C6—C5—H5	120.3
N1^i^—Co1—N1	174.5 (3)	C5—C6—C5^i^	119.7 (11)
N1^i^—Co1—N4	87.26 (16)	C5—C6—H6	120.2
N1—Co1—N4	87.26 (16)	C5^i^—C6—H6	120.2
N4—Co1—Cl1	93.89 (5)	N3—C7—H7A	108.5
N4—Co1—Cl1^i^	93.89 (5)	N3—C7—H7B	108.5
Co1—O1—H1	124.3	N3—C7—C8	115.1 (8)
Co1—O1—H1^i^	124.346 (2)	H7A—C7—H7B	107.5
H1—O1—H1^i^	111.3	C8—C7—H7A	108.5
N2—N1—Co1	119.7 (4)	C8—C7—H7B	108.5
C1—N1—Co1	133.5 (5)	C7—C8—H8A	109.1
C1—N1—N2	103.9 (6)	C7—C8—H8B	109.1
N1—N2—C3	120.5 (5)	C7—C8—C9	112.3 (9)
C2—N2—N1	113.6 (6)	H8A—C8—H8B	107.9
C2—N2—C3	125.9 (6)	C9—C8—H8A	109.1
C1—N3—C2	108.0 (6)	C9—C8—H8B	109.1
C1—N3—C7	126.9 (7)	C8—C9—H9A	107.9
C2—N3—C7	125.1 (6)	C8—C9—H9B	107.9
C4^i^—N4—Co1	121.2 (4)	C8—C9—C10	117.6 (11)
C4—N4—Co1	121.2 (4)	H9A—C9—H9B	107.2
C4^i^—N4—C4	117.6 (8)	C10—C9—H9A	107.9
N1—C1—N3	111.8 (6)	C10—C9—H9B	107.9
N1—C1—H1A	124.1	C9—C10—H10A	109.5
N3—C1—H1A	124.1	C9—C10—H10B	109.5
N2—C2—S1	129.8 (6)	C9—C10—H10C	109.5
N2—C2—N3	102.7 (6)	H10A—C10—H10B	109.5
N3—C2—S1	127.5 (6)	H10A—C10—H10C	109.5
N2—C3—H3A	109.1	H10B—C10—H10C	109.5
N2—C3—H3B	109.1		
			
Co1—N1—N2—C2	162.7 (5)	C1—N3—C2—N2	−1.2 (8)
Co1—N1—N2—C3	−17.8 (8)	C1—N3—C7—C8	83.9 (10)
Co1—N1—C1—N3	−160.2 (5)	C2—N2—C3—C4	−111.9 (8)
Co1—N4—C4—C3	3.1 (7)	C2—N3—C1—N1	1.0 (8)
Co1—N4—C4—C5	−178.5 (6)	C2—N3—C7—C8	−94.5 (11)
N1—N2—C2—S1	−178.3 (6)	C3—N2—C2—S1	2.3 (11)
N1—N2—C2—N3	1.1 (8)	C3—N2—C2—N3	−178.4 (6)
N1—N2—C3—C4	68.7 (8)	C3—C4—C5—C6	175.3 (6)
N2—N1—C1—N3	−0.3 (8)	C4^i^—N4—C4—C3	−176.9 (7)
N2—C3—C4—N4	−58.7 (8)	C4^i^—N4—C4—C5	1.5 (6)
N2—C3—C4—C5	122.8 (8)	C4—C5—C6—C5^i^	1.5 (6)
N3—C7—C8—C9	−72.6 (12)	C7—N3—C1—N1	−177.6 (7)
N4—C4—C5—C6	−3.1 (12)	C7—N3—C2—S1	−3.1 (11)
C1—N1—N2—C2	−0.5 (8)	C7—N3—C2—N2	177.5 (7)
C1—N1—N2—C3	179.0 (6)	C7—C8—C9—C10	−168.6 (12)
C1—N3—C2—S1	178.2 (6)		

Symmetry code: (i) −*x*+1, *y*, −*z*+1/2.

### Hydrogen-bond geometry (Å, º)

**Table:**

*D*—H···*A*	*D*—H	H···*A*	*D*···*A*	*D*—H···*A*
O1—H1···Cl1^ii^	0.84	2.24	3.075 (4)	171
C1—H1*A*···O1^iii^	0.95	2.69	3.429 (7)	135
C3—H3*A*···Cl1	0.99	2.55	3.360 (8)	138

Symmetry codes: (ii) −*x*+1, −*y*+1, −*z*; (iii) −*x*+1, −*y*+1, −*z*+1.
