# Supplementary material for: Crystal structure and spectroscopic properties of aqua­dichlorido­{1,1′-[(pyridine-2,6-diyl-κN)bis(methyl­ene)]bis­(4-butyl-4,5-di­hydro-1H-1,2,4-triazole-5-thione-κN 2)}cobalt(II)
Source: Acta Crystallogr E Crystallogr Commun. 2020 Oct 16;76(Pt 11):1757–61. doi: 10.1107/S2056989020013547 (PMC7643238; doi:10.1107/S2056989020013547)
Supplement: Supplementary file 3 [file e-76-01757-sup2.pdf]

Captions and Figures S1-S4 for jy2002

Figure S1

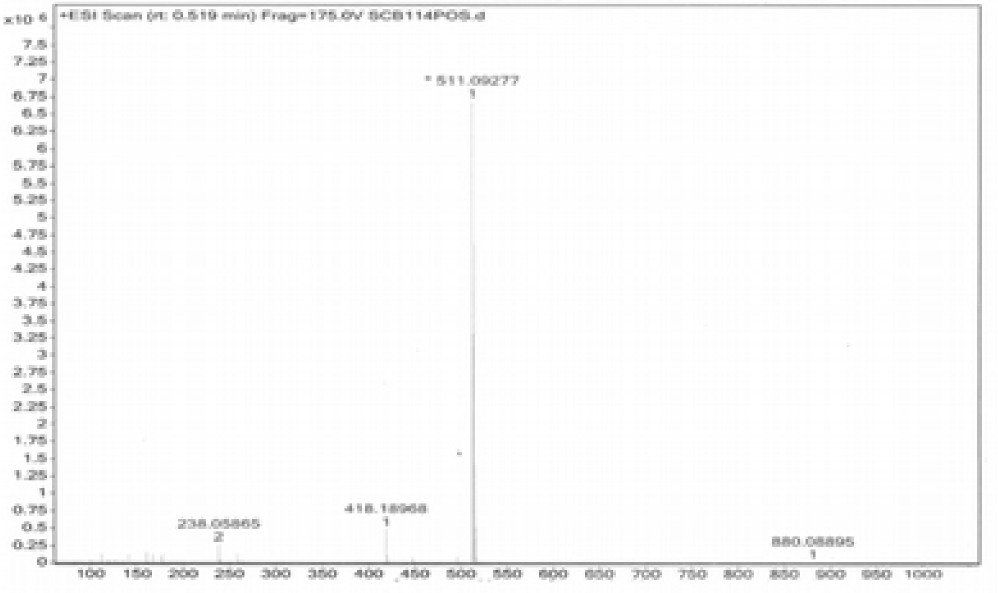

High-resolution electrospray mass spectrum of **8** in positive ion mode indicating loss of water and Cl^{-} ligand from the metal complex.

Figure S2

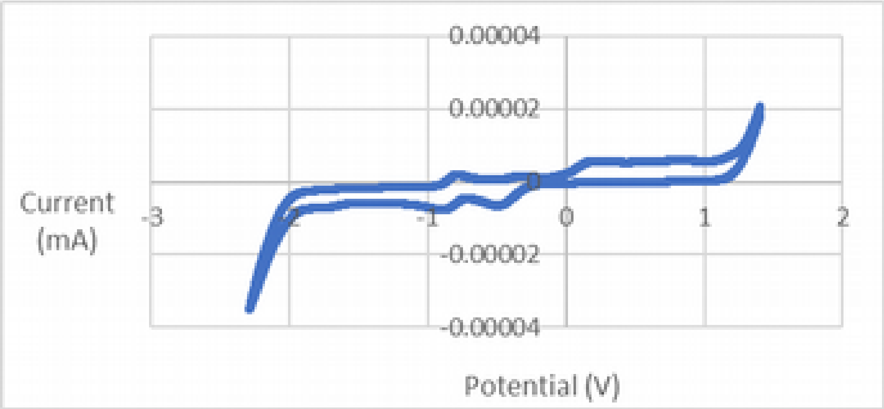

Cyclic voltammogram of **7** (2.0 m*M* in DMSO) indicating the redox-active nature of **7**.

Figure S3

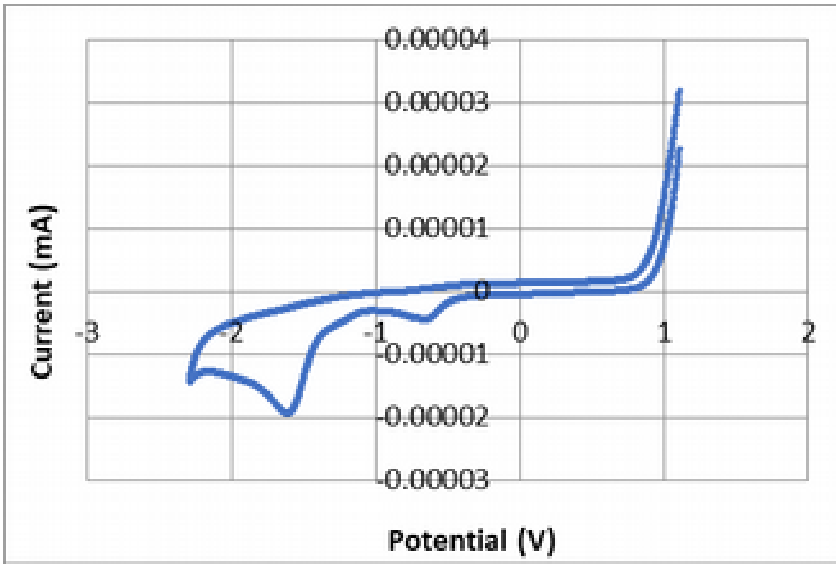

Cyclic voltammogram of **8** (2.0 m*M* in DMSO) indicating the redox-active nature of **8**.

Figure S4

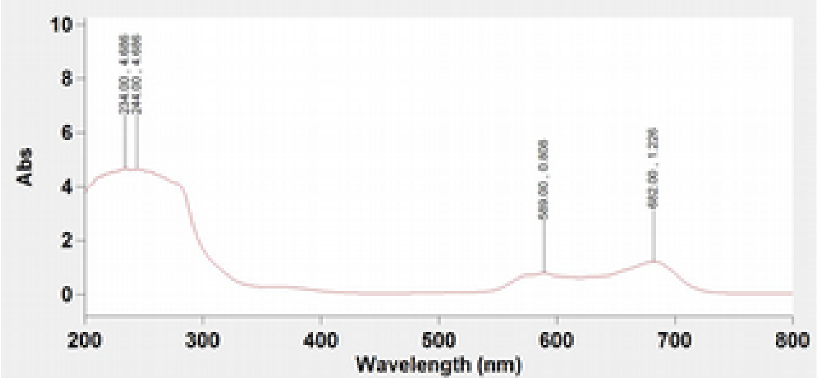

Electronic absorption spectrum of **8** in acetonitrile (1.89 m*M*).
